# Delayed diagnosis of bacterial cervical lymphadenitis in the tropics: a case report

**DOI:** 10.1186/s13256-023-03773-9

**Published:** 2023-02-09

**Authors:** Oladosu Olaniyi, Ojo Ayotunde, Thomas Christabel, Odediran Idowu

**Affiliations:** 1 Outpatient Department, Roding Medical Center, Lagos, Nigeria; 2 Renal Dialysis Center, Ikeja, Lagos, Nigeria; 3 Lagos State University Teaching Hospital, Ikeja, Lagos, Nigeria; 4 Beachland Specialist Hospital, Mowe, Ogun Nigeria

**Keywords:** Cervical, Lymphadenitis, Neck swelling, Fine needle aspiration cytology (FNAC), Lymphadenopathy, Delayed diagnosis

## Abstract

**Background:**

Lymphadenopathy refers to any disease process involving lymph nodes that are abnormal in size and consistency. There are multiple etiologies in the setting of a diseased lymph node, including conditions of infection, autoimmune diseases, and neoplasia. Lymphadenitis is a term that refers to lymphadenopathies that are due to inflammatory processes and can represent an acute bacterial infection resulting from streptococcal pharyngitis or a prior viral upper respiratory infection. Cervical lymphadenopathy refers to nodal swelling in the neck region. While cervical lymphadenitis is a common clinical finding in the setting of a transient response to a benign local or generalized infection, it may evade detection sometimes and thus account for a percentage of misdiagnosis or delayed diagnosis in the tropics. This case report is aimed at increasing the awareness about the presentation of bacterial cervical lymphadenitis and how it can sometimes mimic the presentation that is typical and regularly found with plasmodiasis. It contributes to improved awareness and high index of suspicion in clinic when dealing with patients in the tropics.

**Case presentation:**

We present a case of delayed diagnosis of bacterial cervical lymphadenitis that initially presented with typical features of malaria from *Plasmodium falciparum*. A 26-year-old Nigerian woman presented to the outpatient department following complaints of a recurring fever of a month’s duration and bilateral neck swelling of about 2 weeks prior to presentation.

**Conclusion:**

In the setting of a busy clinic, details are easily missed and salient features in the presentation of a patient that are needed for accurate diagnosis and management could go unrecognized. Hence, this case report highlights the importance of proper examination particularly of lymph nodes and use of different diagnostic modalities for the exact diagnosis of disease.

## Introduction

Lymph nodes are oval-shaped organs of the immune system. They have a wide distribution throughout the body and are linked by lymphatic conduits called ducts. The body has about 600 lymph nodes, and 10% (60–70) of these nodes are situated in the head and neck region [1]. Lymphadenopathy is said to be any abnormality in the size, consistency, and number of lymph nodes. This occurs when there is an invasion or proliferation of inflammatory cells in the lymph nodes. It is a common clinical presentation in outpatient departments while being a source of concern for patients and clinicians alike. Many times, the causes of lymphadenopathy remain undiagnosed in a large number of patients. Usually, these causes can be infectious or noninfectious.

This paper presents a case report in which a patient had a delayed diagnosis of cervical lymphadenitis. While there are numerous documented causes of febrile illness (recurrent), we opine that thorough data gathering combined with detailed physical examination findings can make all the difference and are key components in achieving diagnostic accuracy in patients. It is also our opinion that, despite the endemicity of malaria in the tropics, which leads to malaria being considered among differential diagnoses, clinicians should be more alert, have a high index of suspicion, and consider lymphadenitis in their diagnoses, as its presentation, as this case report reveals, could mimic malaria-like symptoms. In our discussion we consider cervical lymphadenopathy due to infectious processes, specifically bacterial bugs, that is, lymphadenitis.

### Bacterial cervical lymphadenitis

While lymph nodes are located throughout the lymphatic system, their concentration in certain areas of the body is explained by their role in filtering extracellular fluid. Generally, a normal-sized lymph node is less than 1 cm in diameter and is said to be diseased if there is any abnormality in the size, consistency, and number of lymph nodes, which is known as lymphadenopathy. In fact, 75% of all lymphadenopathies are localized, with more than 50% found in the head and neck area, and cervical lymph nodes are more common than lymphadenopathies in other lymphatic regions [2]. Lymphadenitis is a term that refers to lymphadenopathies that are due to inflammatory processes and can represent an acute bacterial infection, in which case it is called bacterial cervical lymphadenitis [3]. It is characterized not only by nodal swelling, but also by pain, skin changes, fever, edema, and/or purulent collections. Cervical lymphadenitis (CL) is characterized by inflammation of one or more lymph nodes in the neck. Usually involved are the anterior cervical, the submandibular, or the posterior cervical nodes. Although reactive inflammation of lymphatic tissue usually occurs in response to an infectious agent, immunologic processes without local infection and certain malignancies may produce a similar histologic or clinical picture.

An unexplained cervical lymphadenopathy is a cause of concern for both physicians and patients because it may be the manifestation of an underlying malignancy and should be accurately diagnosed as early as possible.

## Case report

A 25-year-old female Nigerian patient presented with chief complaints of recurrent fever of 1 month duration and bilateral neck swelling. Fever was described as low grade, continuous, associated with chills and rigor, and relieved only by the use of paracetamol and nonsteroidal anti-inflammatory drugs (NSAIDs). She also noticed bilateral anterior neck swellings about 2 weeks prior to presentation. Swelling was gradual in onset, not painful, and about the size of a peanut when first noticed with no significant increase in size afterwards. Swelling remained the same with swallowing and without any pressure symptoms. She denied any history of cough or contact with a person with chronic cough, and had no history of hoarseness, dysphagia, odynophagia, or stridor. There were other associated symptoms of night sweats, recurrent headaches, nausea, anorexia, and weight loss of about 5 kg at the time of presentation.

Prior to her visit at our outpatient department, she has had multiple medical consultations and multiple courses of antimalarial given over the 1 month period preceding her visit. However, her symptoms were persistent with no resolution. She had no significant past medical or surgical history. History of medications used prior to presentation included three courses (at varying points of illness) of artemether and lumefantrine combination, oral (PO) amoxicillin, PO clotrimazole, PO cotrimoxazole, and PO cefuroxime.

Physical examination findings revealed a young woman who was febrile with bilateral neck lump located at the bases of both the right and left anterior triangles of the neck, both measuring about 2 cm by 4 cm. Lumps were mildly tender and attached to underlying structure but not to the overlying skin. Vital signs at presentation: temperature 37.8 °C, pulse rate 96 beats per minute, blood pressure: 112/78 mmHg. There were no significant neurological, respiratory, cardiovascular, or abdominal findings.

Preliminary laboratory investigations were essentially normal aside from an elevated erythrocyte sedimentation rate (ESR) value.

Complete blood count:

**Table:** Hemogram parameters of the patient

	Test	Result	Reference range
1	PCV	34%	36–48%
	WBC	4900	4000–11,000
2	Neutrophils	59.3%	50–62%
3	Lymphocytes	28.4%	25–36%
4	Eosinophils	0.7%	1–4%
5	Monocytes	11.1%	2–8%
6	Basophils	0.5%	0.5–1%

**Table:** 

	Test	Result	Reference range
1	Malaria parasite film	Scanty parasite seen	
2	ESR	44 mm/hour	1–25 mm/hour
3	HIV I and II rapid screen	Negative	
4	HBsAg	Negative	

*PCV* Packed cell volume, *WBC* White blood cells,* ESR* Erythrocyte sedimentation rate, *HBsAg* Hepatitis B surface antigen, *HIV* human immunodeficiency virus

An assessment of tuberculous adenitis was made to keep in view T-acute lymphoblastic leukemia.

The following further investigations were done:

Mantoux test—no induration after 72 hours. Chest X-ray—normal radiograph.

Neck ultrasonography scan (USS): bilateral septated complex cystic masses measuring 3.86 by 2.31 cm and 3.19 cm by 1.31 cm in the right and left lateral region close to the cervical vascular bundles.

The patient was referred to a surgeon for further review, evaluation, and possible biopsy of the neck masses. A fine needle aspiration biopsy was opted for and done due to the peculiarity/difficult anatomy of neurovasculature in the neck region.

Fine needle aspiration cytology (FNAC): smear of aspirates showed sheets of small- to medium-sized lymphocytes, neutrophils, and few immunoblasts. Also seen are macrophages that are entrapped within fibrillary strands. No atypical cell was seen.

On the basis of these findings, an assessment of acute bacterial lymphadenitis was made and the patient was started on a course of PO levofloxacin for 2 weeks. The enlarged lymph nodes gradually regressed over the course of treatment, and on subsequent follow-up, the patient became symptom-free and has remained so.

## Discussion

In their submission on tropical fevers, Singhi *et al*. [4] described and attested to the difficulty experienced in arriving at specific diagnoses and delay in early empirical treatment posed by the overlapping nature of clinical presentations.

Typically, whether in isolation or as a group, constitutional symptoms such as fever, malaise, headaches, and joint aches are usually the first signals that may herald the onset of infectious diseases, and as a result, the possible diagnoses a clinician is faced with in such a situation can conflict with one another. It will then take more than superficial data gathering to properly assess such patients in the tropics. It is important to carry out a thorough physical examination with a high index of suspicion before an accurate assessment is made and a successful treatment option is formulated for patients.

Unfortunately, the endemicity of malaria in this part of the world has, for the most part, created a bias for malaria diagnosis among clinicians and the willing acceptance of this diagnosis by the patients.

To avoid a diagnostic dilemma, both the clinician and the patient have specific roles to play in the diagnostic process. McDonald *et al*. [5] elaborated on the role of patients in improving their own diagnosis, as well as how suboptimal communication can be a recipe for misdiagnosis or delayed diagnosis. In their review, they went on to describe diagnosis as the troubleshooting stage of care that arises in response to symptoms of a problem or routine screening, while treatment involves formulating and implementing the care plan, after problem identification. This thus implies that quality and appropriate care can be given to patients only when these two categories of care are in synchronization.

Like other causes of tropical fevers, acute bacterial cervical lymphadenitis is often missed on the part of healthcare providers in sub-Saharan Africa due to the numerous conditions that can present with fever. In the case reported, there was a diagnostic dilemma seeing as the patient had, without intending it, swayed the focus of the clinician to a limited number of differential diagnoses—by stressing on the symptom of recurrent fever associated with chills, which she saw as more disturbing, and by being less emphatic on the other symptoms of drenching night sweats, progressive weight loss, and bilateral anterior neck masses that would have allowed the clinicians to probe further. Most times, the patient does not necessarily know what information is valuable for diagnosis, or when to be concerned that a diagnosis is off track [5]. This submission presents a stereotypical conundrum that every doctor working in the setting of an outpatient clinic must battle with on a daily basis. The clinician must (at all times) strive to debunk the common notion of “malaria” as the sole cause of febrile illnesses in the mind of the patient, knowing that the patient is under their care. This is even more important when first-line antimalarial combination therapy medications fail to resolve the patient’s symptoms before presentation. The pathophysiology of infections, whether viral, bacterial, or parasitic, involves both innate and adaptive immune responses with the release of inflammatory cytokines, which mediates the aforementioned constitutional symptoms. Hence, the role of a well-taken history and physical examination cannot be overemphasized, as these are critical components of making a proper diagnosis from the dilemma posed by constitutional symptoms and the vagueness of some patients’ first complaints. In the setting of acute bacterial cervical lymphadenitis, obtaining a history of recent upper respiratory tract symptoms prior to or during presentation is important in the proper evaluation of patients and could be the pointer in determining the etiology of acute lymphadenitis [3]. While clinicians must demonstrate a high index of suspicion during evaluation, to improve the safety and quality of care, the patient must play a role in the diagnostic process. Relevant investigations should be requested, especially a complete blood count, blood film for malaria parasites, assay of inflammatory markers such as erythrocyte sedimentation rate (ESR) and C-reactive protein (CRP), chest X-ray, ultrasonography of the neck and/or involved lymph nodes, sputum analysis for acid-fast bacilli, Quantiferon TB Gold, or GeneXpert, and other relevant investigations depending on the differential diagnoses in view. A fine needle aspiration cytology (FNAC) or excisional biopsy of affected lymph nodes should be considered too [6]. In the case reported, fine needle aspiration cytology was preferred to avoid inadvertent injury to the neurovascular structures around the neck region.

In addition, the ease with which antibiotic medications can be procured over the counter by patients has made them readily accessible and at risk of overuse or abuse in the developing world. Ventola [7] wrote about how the efficacy of antibiotics has been endangered by rapid emergence of resistant bacteria worldwide owing to abuse by patients. Also, antibiotic abuse and, ultimately, resistance is assisted in 30–50% of cases by incorrect prescription from clinicians through incorrect treatment indication or choice of agent or duration of antibiotic therapy [8, 9]. This is corroborated in the case reported wherein the index patient had self-prescribed and prescribed antibiotics without resolution of symptoms until the eventual resolution of her symptoms with PO levofloxacin, which is hardly subject to abuse/misuse. Antibiotic resistance is global and can place a substantial health and economic burden on healthcare systems and population worldwide.

## Conclusion

While bacteria constitute one of the common causes of lymphadenitis and initial presentation could mimic common febrile illnesses such as malaria, this paper suggests that, during the assessment of such patients, clinicians should be alert and have a high index of suspicion to enable an early diagnosis. Key factors that would make early diagnosis possible include, but are not limited to, a thorough physical examination regardless of the busyness that typifies most outpatient clinics. Diagnostic errors mete out a high cost to patients and their families, and to professional caregivers [5]. Hence, an early diagnosis will help reduce the burden of frequent hospital visits by patients and reduce expenses [10]. In the evaluation of infectious lymphadenitis, a useful strategy for the diagnosis will include collection of lymph node specimens followed by histological analysis when possible. Drug therapy is effective and well tolerated once diagnosis is made.

It is important for clinicians to be aware of the clinical manifestations and specific etiology of bacterial lymphadenitis as well as the diagnostic approaches and therapeutic options currently available. An evidence-based algorithm should be formulated to guide clinicians in deciding which diagnostic modality or empiric treatment to administer once diagnosis is made. A good follow-up culture is also required to monitor the need for additional diagnostic tests in the setting that a patient fails to respond to appropriate initial therapy.

## Data Availability

Data sharing not applicable to this article as no datasets were generated or analyzed during the current study.
